# An artificial sensory neuron with visual-haptic fusion

**DOI:** 10.1038/s41467-020-18375-y

**Published:** 2020-09-14

**Authors:** Changjin Wan, Pingqiang Cai, Xintong Guo, Ming Wang, Naoji Matsuhisa, Le Yang, Zhisheng Lv, Yifei Luo, Xian Jun Loh, Xiaodong Chen

**Affiliations:** 1 Innovative Center for Flexible Devices (iFLEX), Max Planck – NTU Joint Lab for Artificial Senses, School of Materials Science and Engineering, Nanyang Technological University, 639798 Singapore, Singapore; 2 Institute of Materials Research and Engineering, Agency for Science, Technology and Research (A*STAR), 138634 Singapore, Singapore

**Keywords:** Electrical and electronic engineering, Sensors and biosensors, Nanosensors

## Abstract

Human behaviors are extremely sophisticated, relying on the adaptive, plastic and event-driven network of sensory neurons. Such neuronal system analyzes multiple sensory cues efficiently to establish accurate depiction of the environment. Here, we develop a bimodal artificial sensory neuron to implement the sensory fusion processes. Such a bimodal artificial sensory neuron collects optic and pressure information from the photodetector and pressure sensors respectively, transmits the bimodal information through an ionic cable, and integrates them into post-synaptic currents by a synaptic transistor. The sensory neuron can be excited in multiple levels by synchronizing the two sensory cues, which enables the manipulating of skeletal myotubes and a robotic hand. Furthermore, enhanced recognition capability achieved on fused visual/haptic cues is confirmed by simulation of a multi-transparency pattern recognition task. Our biomimetic design has the potential to advance technologies in cyborg and neuromorphic systems by endowing them with supramodal perceptual capabilities.

## Abstract (web summary)

Designing bioinspired perceptual system remains a challenge. Here, the authors report a bimodal artificial sensory neuron, integrating a resistive pressure sensor, a perovskite-based photodetector, a hydrogel-based ionic cable, and a synaptic transistor, to implement the visual-haptic fusion for motion control and patterns recognition.

## Introduction

When interacting with the real, dynamic world, biological systems always outshine their electronic counterparts due to their sophisticated sensorimotor skills^1–3^. Emulating the functionality and/or structuralism of the natural system would intrinsically address unmet needs in current digital systems^4–6^. Although bioinspired systems using silicon-based circuits and software have realized some complicated and dexterous sensorimotor functions^7–9^, their efficiency still suffers when data size increases due to centralized and sequential operation. In contrast, the biological systems are essentially run on distributed computing paradigm, whose superior fault tolerance and power efficiency are inherent in the adaptive, plastic, and event-driven network of sensory neurons^10,11^. Therefore, emulating the biological processes from the level of sensory neuron would fundamentally achieve biological perceptual capabilities.

**Fig. 1:**
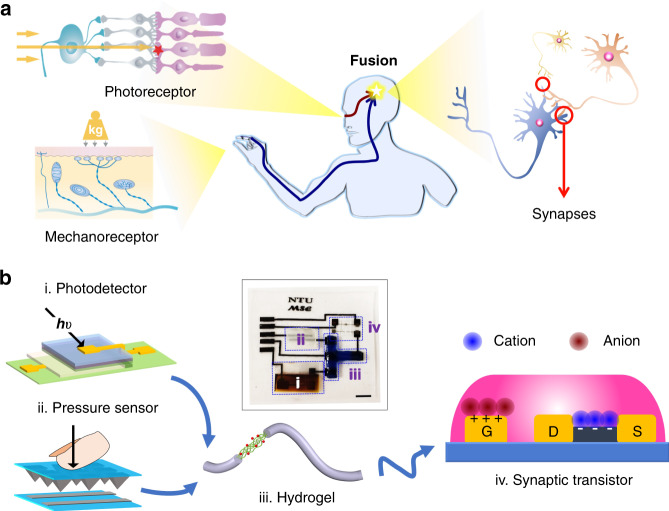
A bimodal artificial sensory neuron with visual-haptic fusion. **a** The visual-haptic fusion by biological neural network. **b** The BASE patch for visual-haptic fusion. Sub-figures i to iv: photodetector, pressure sensor, hydrogel (dyed by 0.04% methylene blue), and synaptic transistor, respectively. Inset: a photograph of the BASE patch. The scale bar is 5 mm.

Artificial sensory neuron development can benefit from an improved understanding of sensory processing in biology. One notable advantage of the biological sensory systems is that they analyze multiple cues, making reactions more reliable than with a unimodal cue. For example, visual and haptic cues are intensively received and perceived in our interactions with the surroundings, and the two cues are closely associated and interpreted in the inferior parietal cortex to provide supramodal spatial ability^12–14^ (Fig. 1a). The neurons in this cortex area subsume both the macrospace of vision and the microspace encompassed by the hand, to avoid misjudgment from environmental complexity such as variances in the object’s pose^9^. The behavioral and psychological experiments also indicate a higher precision of appreciating an object when combining the two cues^9,15^.

Artificial sensory neurons/synapses with either haptic or visual modalities^16,17^, have been achieved for applications such as pattern recognition^18,19^ and muscular contraction control^20^. However, supramodal perceptual capabilities that can increase the reliability and accuracy of these machineries are still absent, possibly due to a lack of platform for mediating multimodal sensory data. Synaptic transistors allow parallel gating of active channels via ions in electrolytes, hence representing a core element for implementing sensory fusion at a neuronal level^21^.

Here, we develop a bimodal artificial sensory neuron (BASE) based on ionic/electronic hybrid neuromorphic electronics to implement the visual-haptic fusion. This BASE unit consists of four core components: resistive pressure sensor, perovskite-based photodetector, hydrogel-based ionic cable, and a synaptic transistor (Fig. 1b). The photodetector and pressure sensor function as the receptors in the retina and skin, respectively, converting external haptic and visual stimuli into electrical signals. The electrical signals from the two sensors are then transmitted through the ionic cable to the synaptic transistors for integration and conversion into a transient channel current, analogous to the biological excitatory postsynaptic currents (EPSC). Bimodal stimuli in closer succession can induce stronger changes in EPSC, which can be used to determine the extent of synchronization between the two. This, in turn, is used to provide multi-dimensional spatial information thereby controlling a biohybrid neuromuscular junction or a robotic hand, mimicking the process of ‘perception for action’^22^. We also design and simulate a matrix of BASE as the feature extraction layer of a perceptron for recognition of multi-transparency alphabetic patterns. The results further confirm that multimodal sensory fusion by BASE can increase the recognition rate (ratio of successful recognition of both the letter and its transparency to total trials) even with a reduced data size. Using BASE for constructing a bioinspired perceptual system would have profound implications for neurorobotics, cyborg systems, and autonomous artificial intelligence.

## Result

### Fabrication and characterization of the BASE

The hybrid neural circuits essentially encompass two artificial sensory channels: the visual and the haptic channel. The visual channel comprises a perovskite-based photodetector, mimicking the photoreceptors in the retina, with a configuration of Zn_2_SnO_4_/PEA_2_MA_2_Pb_3_I_10_/Poly[bis(4-phenyl) (2,4,6-trimethylphenyl) amine] (PTAA)/Au on PET/indium tin oxide (ITO)-coated substrate. 2D perovskite — PEA_2_MA_2_Pb_3_I_10_ is stable in ambient environment for reliable and repeatable photo-detection^23,24^ (Supplementary Fig. 1). The haptic channel comprises a pressure sensor that incorporating microstructures in the top carbon nanotube (CNT)-coated poly (dimethylsiloxane) (PDMS) layer^18,25^ (Supplementary Figs. 2 and 3). When a pressure is loaded on this layer, it forms a resistive pathway with the electrodes on the bottom layer. An increase in pressure increases the contact area and therefore decreases the resistance between the top CNT film and the bottom electrodes. The polyvinyl alcohol (PVA) hydrogel-based ionic cables represent the ionic transmission pathway as the axon in an afferent nerve, which carry information from the two artificial sensory channels to the electrolyte gated synaptic transistors for further integration and processing^26^. The electrolyte gated synaptic transistors are fabricated as shown in Supplementary Fig. 4. The exponential relaxation of ions in the electrolyte after an applied voltage stimulus on the gate (as the presynaptic terminal) explains the slow decay of channel currents (as the EPSC) based on electrostatic coupling^27^. Such decay properties have been investigated for mimicking some essential synaptic plasticity — the basic neurological principle underling learning and memory^28–31^.

**Fig. 2:**
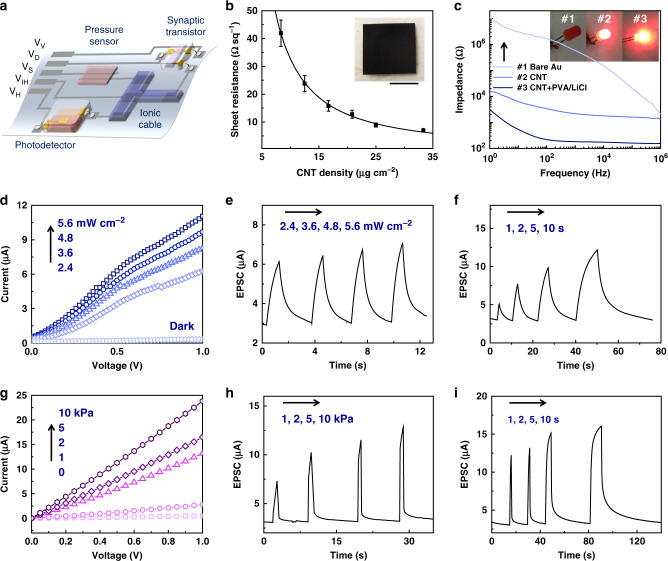
Characteristics of visual and haptic sensory channel illustrating the bimodal sensing capability. **a** The schematic diagram of the BASE for visual-haptic fusion (the sensors are encapsulated by PDMS). **b** The sheet resistance of the CNT electrodes with different density. Inset: the digital image of the test sample with density of 25 μg cm^−2^. The error bars are the standard deviations. The scale bar is 1 cm. **c** The impedance of the PVA hydrogel rod contacted with different electrodes. Inset: the LED connected to different electrodes that are connected with the PVA hydrogel. The drive voltage is 3.0 V with a frequency of 1 kHz. Visual sensory channel characterizations: **d** IV characterizations; **e** the EPSC responses to four different intensities (duration: 1 s); **f** the EPSC responses to four different durations (intensity: 4.8 mW cm^−2^). Haptic sensory channel characterizations: **g** IV characterizations; **h** the EPSC responses to four different intensities (duration: 1 s); **i** the EPSC responses to four different durations (intensity: 2 kPa).

These components are wired by the CNT electrodes fabricated through printing-filtration-transferring processes (Supplementary Figs. 5 and 6) on PDMS films, and such ionic/electronic hybrid circuits are illustrated in Fig. 2a. Three input terminals are connected to the voltage supply for photodetector (*V*_V_), pressure sensor (*V*_H_), and global inhibitory input (*V*_IH_), respectively. The energy consumption of the BASE could decrease down to <0.05 μW by introducing the global inhibitory input (Supplementary Fig. 7). Two output terminals (*V*_D_ and *V*_S_) are connected to the drain and source electrodes of the synaptic transistor, and a voltage bias (*V*_DS_ = 0.5 V) is applied between them for measuring the EPSC. The lowest sheet resistance of 7.0 Ω sq^−1^ of such CNT electrodes has been obtained with a density of 33 μg cm^−2^ (Fig. 2b), indicating decent conductivity for applying/collecting electronic signals from each terminal. Moreover, such CNT electrodes exhibit much lower interfacial impedance (*f* < 1 kHz) with hydrogel than that of gold electrodes (Supplementary Fig. 8), enabling effective electronic–ionic current transduction at the electrode-hydrogel interfaces. Figure 2c shows the impedance characterizations of PVA hydrogel rods bonded to gold and CNT electrodes, respectively, and the latter is almost three orders of magnitude lower than the former. The ionic cable is then fixed on the CNT electrodes by using the instant tough bonding method^32^, maintaining low interfacial impedance (Supplementary Fig. 9).

The information flow in such circuits starts from triggering specific sensors in response to an external stimulus. For artificial visual receptor, the resistance of the photodetector would decrease with an increase in incident light intensity as shown by the current–voltage (IV) measurement (Fig. 2d). The voltage supply (*V*_V_) thus could induce an ion flux through the ionic cable due to the reduced resistance of the sensor. As the other side of the hydrogel is connected to the gate of the synaptic transistor, the accumulation of the ions would electrostatically couple an EPSC through the semiconducting channel of the transistor. The peak EPSCs are influenced by not only the magnitude of the pressure stimulus (Fig. 2e) but also the duration of the light stimulus (Fig. 2f). A similar trend can be found also in the artificial haptic receptor (Fig. 2g–i). Although slight differences between the output characteristics of the two artificial receptors are observed — the pressure sensors show a better linearity and faster responsivity than the photodetectors, the output range of the two kinds of receptors are very similar.

### Motion control based on visual-haptic fusion

**Fig. 3:**
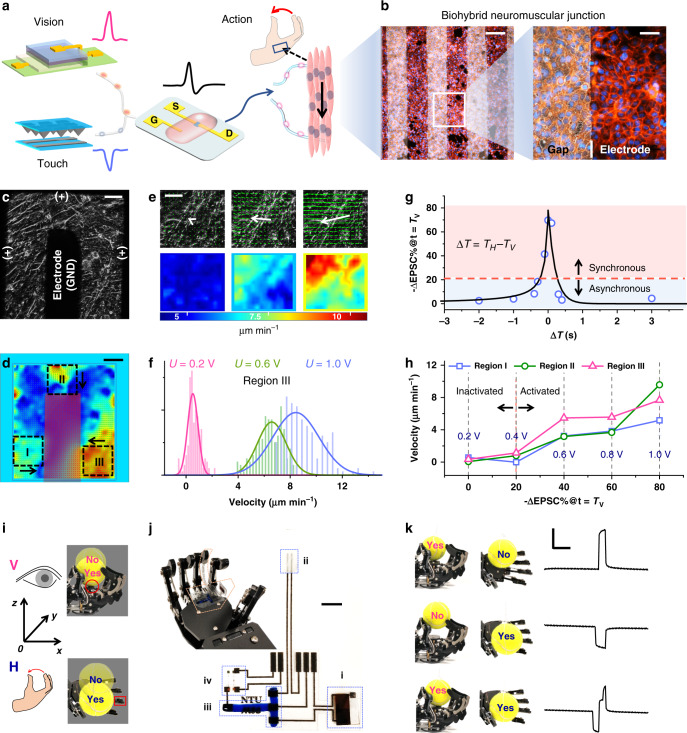
The visual-haptic fusion based on BASE patch for motion control. **a** The schematic of visual-haptic fusion for muscle actuation. **b** Confocal fluorescent imaging of biohybrid neuromuscular junction. The scale bar is 200 μm (left) and 50 μm (right), respectively. **c** Phase contrast image of a target-area (7.3 × 7.3 mm^2^) in the biohybrid neuromuscular junction. The scale bar is 1 mm. **d** Representative speed magnitude mapping of the target-area under 1.0 V stimulation. Region I to III indicate three robust regions of interest. The arrows indicate the dominant myotubes motion direction. The scale bar is 1 mm. **e** Representative mapping of the velocity vector (upper row) and speed magnitude (|*v*|, lower row) in Region III, under stimuli of 0.2 V, 0.6 V, and 1.0 V bias. The white arrows indicate the mean velocity vector. The scale bar is 200 μm. **f** Histogram plot of the motion velocity under 0.2 V, 0.6 V, and 1.0 V. Solid curves: Gaussian fitting of the histogram. **g** Plot of the relative change of ∆EPSC% as a function of the time interval (Δ*T*). The voltage applied on the photodetector and pressure sensor is positive (*V*_V_ = 1.0 V) and negative (*V*_H_ = −1.0 V), respectively. In all, 20% is defined as the criterion of synchronization. **h** The mean speed of region I to III in response to different EPSC changes. The texts in blue indicate the applied voltage converted from ΔEPSC%. **i** The “YES” and “NO” positions inferred by visual (top, pink) or haptic (bottom, blue) feedback. If the ball could be held by the robotic hand based on one sensory feedback, then the position is annotated as “YES” otherwise “NO”. **j** The modified BASE patch on the robotic hand and the magnified image of the BASE patch. The scale bar is 5 mm. **k** The *ΔEPSC* of the BASE with the ball at different positions (*V* = YES, *H* = NO; *V* = NO, *H* = YES; *V* = YES, *H* = NO) through the exploration process. The scale bars are 1 μA and 1 s for *y*-axis and *x*-axis, respectively.

Next, we have designed and fabricated a biohybrid neuromuscular junction (BNJ) to transmit signals from the BASE and to innervate the skeletal myotubes, mimicking body motion control based on visual-haptic fusion (Fig. 3a). The BNJ consists of interdigital electrodes and cultured skeletal myotubes (Fig. 3b). To reduce the impedance between the gold electrode and the myotubes, a layer of Polypyrrole (PPy) has been coated onto the gold electrode by electroplating. As the conducting polymers can transfer charge both by electronic and ionic mechanisms^33,34^, thus the impedance magnitude has been greatly reduced from ~1 MΩ to ~3 kΩ at *f* = 1 Hz (Supplementary Fig. 10). C2C12 myoblast have been seeded after fibronectin functionalization of the BNJ surface. Myoblasts adhere and spread evenly upon overnight incubation in the seeding medium (Fig. 3b). To induce formation of myotubes, the cells were incubated in the differentiation medium for another 5 days. The voltage stimulation can then be applied on the BNJ as shown in Fig. 3c. Particle image velocimetry (PIV) analysis confirms the adhesion between electrodes and cells, and several areas of myotubes can be effectively activated by applying 1.0 V stimulation within 1 min (Fig. 3d). We intentionally choose three regions (region I to III) which are apparently active under electric field. Generally, the myotubes would shrink and show a motion pattern directing from the positive terminal to the ground (Supplementary Fig. 11). Region III is used to illustrate the stimulation intensity dependent contraction property (Fig. 3e), and a clearly positive correlation between voltage intensity and activeness (indicated by mapping of the magnitude of velocity) can be observed. The histogram of *x* component of velocity has also quantitatively verified such conclusion (Fig. 3f).

To localize a nearby object, we might glance by eye (*t* = *T*_V_) and explore by finger (*t* = *T*_H_). The eye-hand coordination enables us to combine the two cues for spatial inference. Then we grasp the object via the contraction on muscle groups of the hand allows us to grasp the object, once its location has been confirmed. Moreover, human beings usually tolerate certain amount of temporal discrepancy between two signals while still perceiving them as being synchronous^35,36^. To mimic such synchronous action, the visual stimulus (~4.8 mW cm^−2^, ~250 ms) and haptic stimulus (~2 kPa, ~350 ms) with different time intervals (∆*T* = *T*_H_–*T*_V_) are applied on the visual and haptic sensory channels of the BASE, respectively. The voltages applied on the two channels (*V*_V_ and *V*_H_) are 1.0 and −1.0 V, respectively. Since both the EPSCs triggered by each of the two stimuli exhibit a gradual decay as demonstrated in Fig. 2, they may affect the response following an immediate subsequent stimulus^37^. Consequently, the EPSC amplitudes (∆EPSC) measured at *t* = *T*_V_ show no obvious change compared with ∆EPSC_V_ (the EPSC amplitude triggered by visual stimulation only) when |∆*T*| > 1 s, while with shorter time intervals, the amplitudes decrease gradually with decreasing |Δ*T*| (Fig. 3g). Here, we set the relative change of ∆EPSC against ∆EPSC_V_ ((∆EPSC-∆EPSC_V_)/∆EPSC_V_, annotated as ∆EPSC%) of 20% as a threshold for determining synchronous (|ΔEPSC%| > 20%) or asynchronous (|ΔEPSC%| ≤ 20%) signals. The ΔEPSC are detected and linearly converted to voltage outputs within the range of 0–1.0 V, which are then sent to the biohybrid neuromuscular junction for myotubes control (Supplementary Fig. 12). Our results show the voltage applied on the BNJ can trigger a perceptible migration when the voltage is >0.4 V (Supplementary Fig. 13). The output voltage is no more than 0.4 V for the asynchronous signals, which cannot trigger the perceptible action of the myotubes (Fig. 3h and Supplementary Movies 1–3). Conversely, the synchronous signals can induce the obvious migration of the myotubes.

**Fig. 4:**
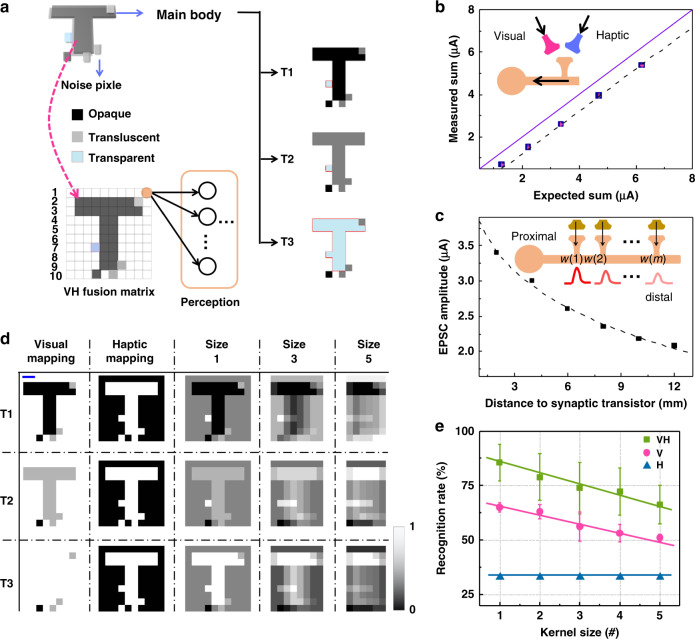
The BASE matrix based visual-haptic fusion for multi-transparency pattern recognition. **a** The multi-transparency alphabetic patterns (top left) and the visual-haptic fusion matrix (bottom left). The sensing data of each pattern obtained from the VH fusion matrix are fed into a perceptron for recognition. Each pixel could be opaque (top right, shown in black), translucent (middle right, shown in gray), or transparent (bottom right, shown in light blue). The patterns are labeled based on the shape of the main body and their transparency. For example, the ‘T1’, ‘T2’, and ‘T3’ denote the ‘T’ shape patterns which are opaque, translucent, and transparent, respectively. **b** The fusion results of two equidistance inputs. Two voltage inputs with different amplitudes are applied to a synaptic transistor through ionic cable with two isometric branches. The expected sum is the arithmetic summation of the EPSCs by individually triggering the two inputs, and the measured sum is the EPSCs by simultaneously triggering the two inputs. **c** The output measured from different inputs that are connected to the synaptic transistor through one common ionic cable. The weight for each input thereby annotated as *w*(1) to *w*(*m*). **d** The normalized mapping results based on unimodal information (visual or haptic) and VH fusion information. n-VH fusion data is merged from every 1, 3, and 5-VH units to 1 synaptic transistor in each line of the VH fusion matrix. The scale bar (in blue) is 5 mm. **e** The recognition rates for the mapping of unimodal and bimodal modes with kernel sizes of 1–5, respectively. The error bars are the standard deviations.

The strategy of fusing visual and haptic feedbacks can also enable robotic motion control and is superior to a unimodal one. A tennis ball located at the proximity of a robotic hand can be noted as “YES” (or “NO”) if it could (or not) deliver either the visual or haptic feedback to trigger the hand closing for catching the ball (Fig. 4i, visual feedback for *z*-axis or haptic feedback for *y*-axis). The ball can only be caught when it is at the “YES” position for both directions. The unimodal feedback could only be used to differentiate “YES” or “NO” at one-dimension, due to the binary states of each sensor. By contrast, a modified BASE patch is able to obtain the spatial information from the two dimensions (Fig. 4j) for guiding a robotic hand. The spatial information is obtained through an exploration process similar as before: (1) triggering a LED on the ball, providing the visual stimulus; (2) half-closing the hand to touch the ball, providing the haptic stimulus. As shown in Fig. 4k, the BASE can generate three types of responses by the exploration process (*ΔT* = 100 ms), when the ball is at three typical positions (*V* = YES, *H* = NO; *V* = NO, *H* = YES; *V* = YES, *H* = YES). In this way, the robotic hand can take more appropriate action (open or close) based on the multi-dimension information. Otherwise, the utilization of one-dimension spatial information might lead to wrong decision for the robotic hand (Supplementary Fig. 14, Tables 1 and 2, Scheme 1, and Movies 4–8).

### Multi-transparency pattern recognition

Subsequently, we design BASE-based sensory fusion matrices to use the fused visual and haptic cues for recognition tasks (Fig. 4a). Multi-transparency alphabetic patterns (a mixture of PDMS and carbon black, Supplementary Figs. 15 and 16) have been used in this work. The alphabetic patterns are labeled based on the shape and transparency of their main body. To verify the robustness of the recognition, 720 alphabetic patterns with different combinations of random noise pixels have been used for recognition (Supplementary Fig. 17). The sensing data from each matrix are then fed into a perceptron with one hidden layer built by MATLAB for the pattern recognition tasks (Supplementary Fig. 18 and Supplementary Training Samples). In this case, this matrix serves as the feature extraction layer of this artificial neural network.

The bimodal fusion matrix and unimodal matrices (optics or pressure) are designed with 10 × 10 pixels. Each pixel of the bimodal fusion matrix contains one photodetector and one pressure sensor (noted as VH unit). In each line, the ten VH units are connected to synaptic transistors through the ionic cable. As shown in Fig. 4b, the transistor can integrate stimuli from the VH unit inducing a joint EPSC current which is dependent on the intensity of the two stimuli and the weight of the input (*w*). Our results show that the summation output (measured sum) from one VH unit is slightly lower than the arithmetic summation (expected sum) of the outputs (Fig. 4b and Supplementary Fig. 19). The weight of the input (*w*(*m*)) is dependent on the distance between the transistor and the VH unit — a gradual decay with an increasing distance (Fig. 4c and Supplementary Fig. 20). Therefore, the integration effect of the synaptic transistor to multiple inputs could be deemed as the integral of the product of the input intensities and their distance-dependent weights. Here, we define the kernel size (*n*) of the feature extraction layer as the numbers of VH units in one row connected to one transistor in series through one common ionic cable (i.e., VH unit *i* to *i* + *n*−1 (*i* = 1, 2, … 10−*n* + 1). Such configuration thereby could implement the convolution-like operation to the input from each VH unit (annotated by *n*-VH, Supplementary Fig. 21). The convolutional layer is the core building block of a convolutional neural network (CNN), which serves as the filter and efficiently extracts the essential features of input. As such, the mapping output (EPSC amplitude from each transistor) of the sensory fusion matrices can be calculated by computer using the extracted device parameters from these results (Supplementary Fig. 22 and Note 1).

To obtain the visual and haptic cues of the multi-transparency patterns, we place the patterns on the arrays for ~1.5 s with a light source (~4.8 mW cm^−2^) turned on at the same time, and thereafter remove the pattern with the light turned off. The normalized sensing data by unimodal matrices and bimodal matrix are shown in Fig. 4d. When mapping the multi-transparency patterns, simply exploiting the optic matrix would lose some essential features, while simply exploiting the pressure matrix could only provide the shape information without the degree of transparency. Only the visual-haptic fusion matrix could extract both the shape and the transparency adequately. Consequently, the recognition rate based on the visual-haptic fusion matrix shows the best outcome as shown in Fig. 4e. Interestingly, although the features of the alphabetic patterns extracted by increasing the kernel size of the matrix would be lost (60% of original data size with *n* = 5) due to the spatial integration, the recognition rate (~66%) by the fusion mode with *n* = 5 is still slightly better than that of the unimodal mode (~65% for the optic matrix with *n* = 1). Such results indicate we can achieve robust recognition by using the VH fusion matrix as the feature extraction layer of an artificial neural network.

## Discussion

In summary, inspired by the supramodal sensory fusion in the sensory nervous system, we developed an ionic/electronic hybrid neuromorphic device that combines optic and pressure stimuli to generate a summed EPSC through the synaptic transistor. Such combined current carries the bimodal information in time-dependent and nonlinear manners, which closely resemble the neuronal behaviors. The fused signal was then used to innervate the skeletal myotubes and provide multi-dimensional spatial information for the robotic hand, successfully mimicking the motion control by using bimodal sensory cues at the cellular level. More interestingly, the bimodal sensory data could be implemented for recognition of multi-transparency alphabetic patterns and exhibits superior performance to unimodal sensory data. The multi-transparency patterns could represent and abstract the core factors of some real scenarios where the transparency of the object could be inferred by visual feedback and the shape or weight could be inferred by tactile feedback.

We have summarized recent achievements in artificial sensory neurons as shown in Table 1. Compared with other works, our BASE can fuse the cues of two sensory modalities and use them for manipulation, recognition, and synaptic emulations. In addition, with the successful incorporation of myotubes as the actuator, all components in the current system are performing functions at the cellular or equivalent level, which is of great potential for assembling the bionic sensorimotor system from the bottom up. As distinct from previous biohybrid robots using direct bias or optogenetic stimuli not representative of actual real-world cues^38–40^, our biohybrid neuromuscular junction can trigger the contraction of myoblasts by visual and/or tactile -like stimulation, hence moving towards biologically relevant sensorimotor systems. Conventional approaches for sensory processing rely on centralized and sequential operations of data, in which the efficiency dramatically decreases with the increase in data size. In contrast, the neuronal-level implementation, incorporating sensing, refining, and processing would eventually achieve the biological advantages of fault tolerance and power efficiency. Although multi-sensory capabilities have been realized in some e-skin systems^41–43^, their sensory processing has always been completed using conventional digital units. As such, mimicking sensory fusion from the neuronal-level could help to build a highly integrated perceptual system to access massive sensory data for improving current cyborg technologies^44,45^ and artificial intelligence^46–48^.

**Table 1:** Summary of state-of-the-art in artificial sensory neurons.

Name	Sensory modality	Actuator & size	Feature action dimension	Recognition pattern	Synaptic emulations
Artificial afferent nerve^19^	Pressure	Insect leg ~3 cm	~1 mm	Braille pattern	Short-term potentiation
NeuTap^18^	Pressure	NA	NA	Braille pattern	Short-term potentiation; dendritic integration
Artificial somatic reflex Arc^49^	Pressure	Electrochemical actuator ~2.5 cm	~1 mm	NA	Neural all-or-none law
Artificial optic-neural synapse^50^	Light	NA	NA	Colour-mixed pattern	Long-term potentiation & depression
Optoelectronic sensorimotor artificial synapse^20^	Light	Polymer actuator ~2 cm	~1 mm	NA	Short-term potentiation
Optoelectronic neuromorphic device^51^	Light	NA	NA	Illuminance gradation pattern	Visual synaptic functions; environment-adaptable perception
This work (BASE)	Pressure & light	Myotubes ~1.5 mm	~5 µm	Multi-transparency pattern	Dendritic integration

## Methods

### Fabrication of the photodetectors

The fabrication processes of the photodetectors are briefed as follows. ITO/PET was chemically etched by reaction of zinc powder with HCl (2 M) for the desired pattern before being cut into 2.5 × 2.5 cm^2^ pieces. The ITO/PET was washed and rinsed with detergent, DI water, and ethanol sequentially. After drying with nitrogen gas, the ITO/PET was secured onto glass substrates and treated in UV-ozone plasma for 15 min. Zn_2_SnO_4_ nanoparticle solution was deposited on prepared ITO substrate by spin coating at 3000 rpm for 30 s. The coated substrates were dried at 100 °C for 10 min, followed by annealing at 150 °C for 2 h. Perovskite precursor (1.2 M) and substrates were preheated to 100 °C, before the hot precursor solution was spin coated onto substrates at 4000 rpm for 30 s, followed by annealing at 100 °C for 15 min. PTAA solution was spin coated on cool perovskite films at 3000 rpm for 60 s. Au electrodes (~100 nm) were thermal evaporated on samples with the size of 0.2 cm × 0.2 cm.

### Fabrication of pyramidal structured PDMS film

Silicon masters with recessed pyramidal microstructure arrays were fabricated using our previous method by photolithography and wet etching process^17^. The mixture of PDMS elastomer and crosslinker in 10:1 (w/w) ratio (Sylgard 184, Dow Corning) were spin coated on PDMS at 800 rpm. The elastomer mixture was degassed in vacuum and cured at 90 °C for 1 h. The films were then sectioned by a scalpel and peeled off from the silicon master.

### CNT deposition through ultrasonic vibrating method

The CNT were sprayed on the microstructured PDMS film through ultrasonic vibrating method (Supplementary Fig. 2a). In brief, 5 μL CNT solution (1 mg/mL) was diluted in 2 mL deionized water to obtain a homogeneous CNT solution of 2.5 μg/mL through stirring. A commercial ultrasonic humidifier was used to generate microdroplets of the CNT solution through a filter screen with hole size of 60 μm. A time controller was used to trigger/stop the generation process. For fabrication, the PDMS films were placed on a hotplate at 120 °C. One fabrication cycle takes 1 min. The CNT microdroplet were generated through the ultrasonic vibrator and sprayed onto the PDMS film for 5 s. It was then dried out to form a CNT film on the electrodes for another 55 s.

### Fabrication of the synaptic transistors

The fabrication of the PVA gated synaptic transistors can be obtained in previous reports^17^. In brief, the patterned ITO electrodes (drain, source, and gate electrodes) with a thickness of ~60 nm were deposited by radio-frequency magnetron sputtering through a stainless steel shadow mask. Then a ~10 nm ITO channel was deposited using the same method. PVA powder was dissolved in 5% CaCl_2_ solution at a concentration of 10 wt%, then heated gently using a hot oil bath for complete dissolution without thermal decomposition of the polymer. Then the PVA gate dielectric was cast onto the channel and gate electrode, and dried at 60 °C for 2 h.

### CNT patterning process

The patterns were designed using PowerPoint software. The Nylon filter membrane (1 μm) was adhered to A4 paper for printing. The designed patterns thus were printed onto the filter membrane. Then the CNT solution was vacuum-filtered on the filter membrane. CNT could only be concentrated on the white areas of the filter membrane, because the micropores of the membrane at the black areas were penetrated with the printing ink which impedes the passing through of solvent. The membrane was then thoroughly rinsed with DI water to remove the residual sodium dodecyl benzene sulfonate (SDBS) and CNT on the black area. Next, the PDMS (10:1) which was heated in the oven for 35 min (60 °C), was used for transferring the CNT pattern by the hot-press approach. The membrane with the CNT pattern face was put onto the half-cured PDMS, and a glass and a 200-g weight were covered onto the membrane for achieving close contact between the membrane and PDMS. The PDMS and the membrane were then heated in the oven for 4 h (60 °C) before separating of them.

### PVA based hydrogel fabrication process

The PVA (Aldrich, MW = 124,000) powder was dissolved in deionized water at a concentration of 10 wt%, then heated gently using a hot oil bath (90 °C) for complete dissolution without thermal decomposition of the polymer. The hot solution was stirred until the polymer was completely dissolved and a clear viscous solution is formed. Then the PVA hydrogel is obtained through three freeze-thaw cycles. To obtain a high ionic conductivity, the PVA hydrogel was immersed in the 1 mol L^−1^ LiCl for 4 h.

### Fabrication of the biohybrid neuromuscular junction

The interdigital Au electrodes with thickness of 70 nm were deposited through thermal evaporation using a shadow mask. Then the Polypyrrole (PPy) was coated onto the Au electrode by electroplating. The PPy was synthesized on the interdigital Au electrodes by anodic oxidation of pyrrole in an electrochemical cell containing 0.42 g pyrrole monomer, p-toluenesulfonic acid (PTSA, 25 mg) dissolved in 30 mL 5% phosphate buffer saline (PBS) at temperature of ~0 °C. Pyrrole was first distilled and kept refrigerated until use. Dissolved oxygen was removed by bubbling the solution with nitrogen for several minutes prior to polymerization. The galvanostatic polymerization was carried out by using Keithley 4200 with the constant current density of 0.8 mA/cm^2^. An Au plate serves as the counter electrode. The solution was kept under stirring by a magnetic stirrer during the polymerization process. After 3 min of electrochemical polymerization, a dense layer of PPy was deposited on the surface of Au electrode. The obtained electrodes were dipped in DI water for three times at 20 min each. C2C12 myoblasts were seeded onto the electrode substrate in the seeding medium (DMEM with 10% fetal bovine serum) overnight. Subsequently, the cells were induced to form myotubes in the differentiation medium (DMEM supplemented with 2% fetal bovine serum and 1 µg/mL insulin) for 5 days, forming the biohybrid neuromuscular junction.

### The recognition simulation

The six basic alphabetic patterns were extended with three transparencies: transparent, translucent, and opaque. Each pattern was added with five noise pixels (40 cases in total) as one multi-transparency pattern for recognition task. The distribution and the transparency of noise pixel were generated by MATLAB. As such, there are 720 multi-transparency patterns in total. The data obtained from the sensory fusion matrix was fed into the two layers of perceptron. There are 10 nodes in the hidden layer and 18 nodes in the output layer corresponding to the 18 labels of patterns. The network was then trained by MATLAB.

### Reporting summary

Further information on research design is available in the Nature Research Reporting Summary linked to this article.

## Supplementary information

SUPPLEMENTARY INFO

Description of Additional Supplementary Files

Movie 1

Movie 2

Movie 3

Movie 4

Movie 5

Movie 6

Movie 7

Movie 8

Reporting Summary

## Data Availability

The data that support the plots within this paper and other finding of this study are available from the corresponding author on reasonable request.

## Code availability

Code from this study is available from the corresponding author upon request.
