# Supplementary material for: An artificial sensory neuron with visual-haptic fusion
Source: Nat Commun. 2020 Sep 14;11:4602. doi: 10.1038/s41467-020-18375-y (PMC7490423; doi:10.1038/s41467-020-18375-y)
Supplement: Supplementary file 1 — SUPPLEMENTARY INFO [file 41467_2020_18375_MOESM1_ESM.pdf]

Supplementary Information

An Artificial Sensory Neuron with Visual-Haptic Fusion

Changjin Wan, *et al.*

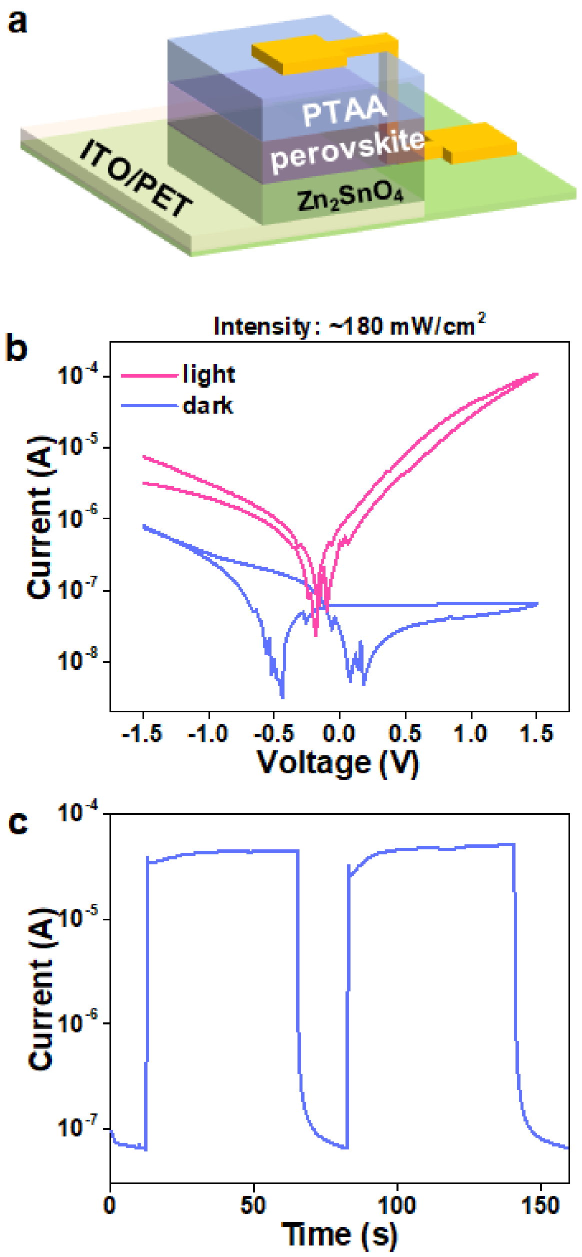

Supplementary Figure 1: Characterizations of the photodetector. a, The layer-by-layer structure of the perovskite based photodetectors. **b,** The I-V curves for the photodetectors under dark and 180 mW cm^{-2} light intensity conditions, respectively. The voltage applied on the ITO electrode swept from -1.5 to 1.5 V. **c,** The I-t curve of the photodetector in response to periodic light pulses with 180 mW cm^{-2} in intensity. Such photodetectors exhibited stable performance with the light being turned on and turned off.

The I-V and I-t tests were measured by semiconductor parameter characterization system (Keithley 4200 SCS). The light source for the characterizations was provided by a solar simulator (HAL-320, ASAHI SPECTRA Co. Ltd). The light intensity was measured by a laser power meter (COHERENT).

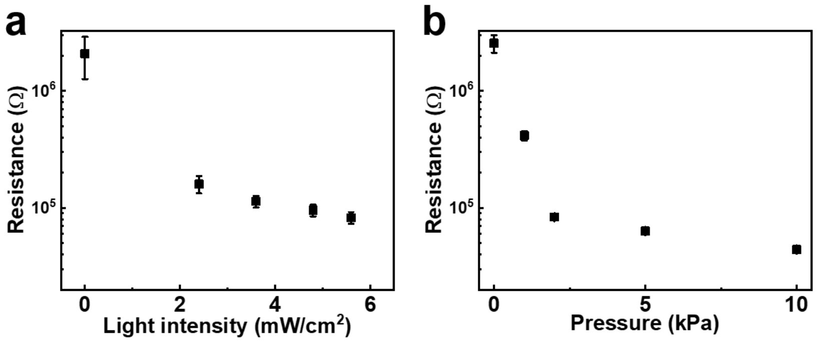

Supplementary Figure 2: The resistances of a, photodetector plotted as function of light intensity and **b,** the pressure sensor plotted as function of pressure, respectively. The resistances of the photodetector and the pressure sensor are measured with different voltages (0-1 V in 0.02 steps, and 0-1 V in 0.05 steps), respectively.

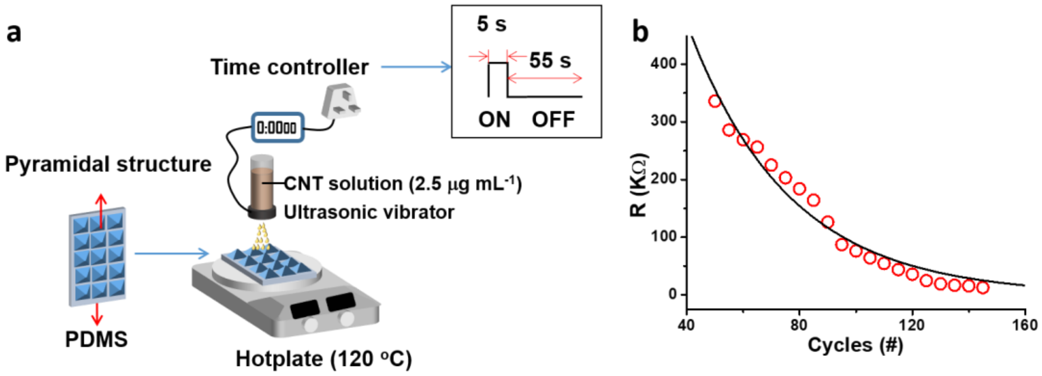

Supplementary Figure 3: The fabrication and characterization of CNT/PDMS based pressure sensitive layer.[1] **a,** The CNT film was sprayed by ultrasonic vibrator on pyramidal microstructured PDMS. **b,** The resistance of the CNT resistors versus time. The resistance can be tuned from ~330 to 12.5 kΩ in ~145 cycles. The red circles are experimental data and the black line is the fitting curve.

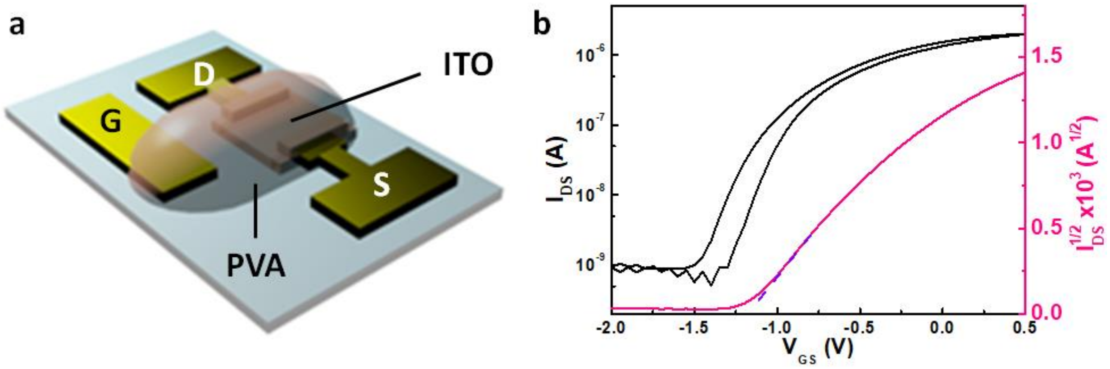

Supplementary Figure 4: The fabrication and characterization of PVA gated ITO synaptic transistors. a, The schematic diagram of the PVA gated synaptic transistors. The fabrication of the PVA gated synaptic transistors can be obtained in previous reports.[2] **b,** The transfer curves of the transistors measured at V_{DS}=1.5 V. The transfer characterizations were measured by semiconductor parameter analyzer (Keithley SC4200). All measurements were done at room temperature with a relative humidity of ~50%. Voltage applied on an in-plane gate can laterally tune the conductance of the ITO channel by three orders of magnitude. A threshold voltage about -1.25 V was calculated from the *x*-axis intercept of the square root of I_{DS}-versus-V_{GS} plot indicating fully depletion mode operation.

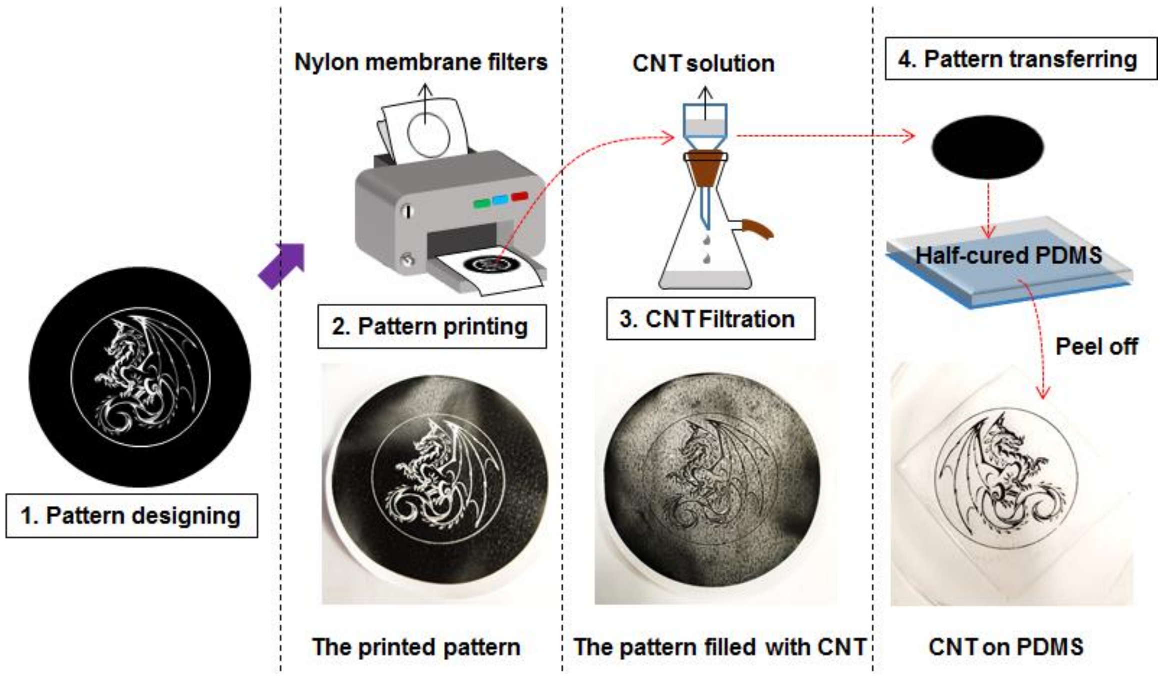

Supplementary Figure 5: CNT electrodes patterning through printing-filtration-transfer (PFT) processes. The patterning of CNT has been described in Method and the preparation of CNT solution was briefed as: CNT powders (Bu-202, Bucky USA) were firstly treated with 1M HNO3 aqueous solution to remove the metallic impurities, followed by filtration with DI water for five times. Next, the aqueous mixture solution with 1 mg mL^{-1} CNT and 2 mg mL^{-1} sodium dodecyl benzene sulfonate (SDBS) was stirred at 1000 rpm for 12 h. Finally, 1 h bath sonication was followed by 1 h probe-type sonication to obtain a homogenous mixture solution.

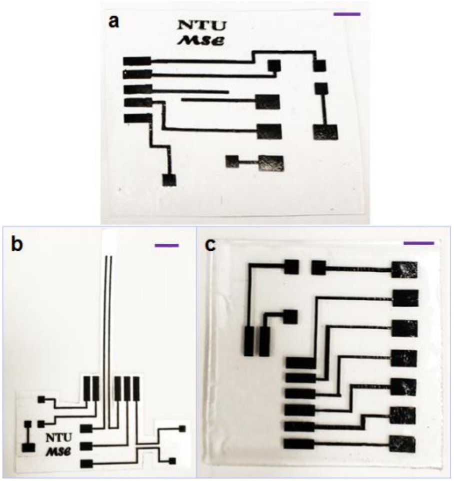

Supplementary Figure 6: The digital images of CNT circuits by using PFT method. a, The basic circuits for visual-haptic integration. **b,** A transformed circuit of the circuit in **a**. **c,** The circuits for the distance-dependent output properties measurement (used in Figure 4d). The scale bar for all cases are 5 mm.

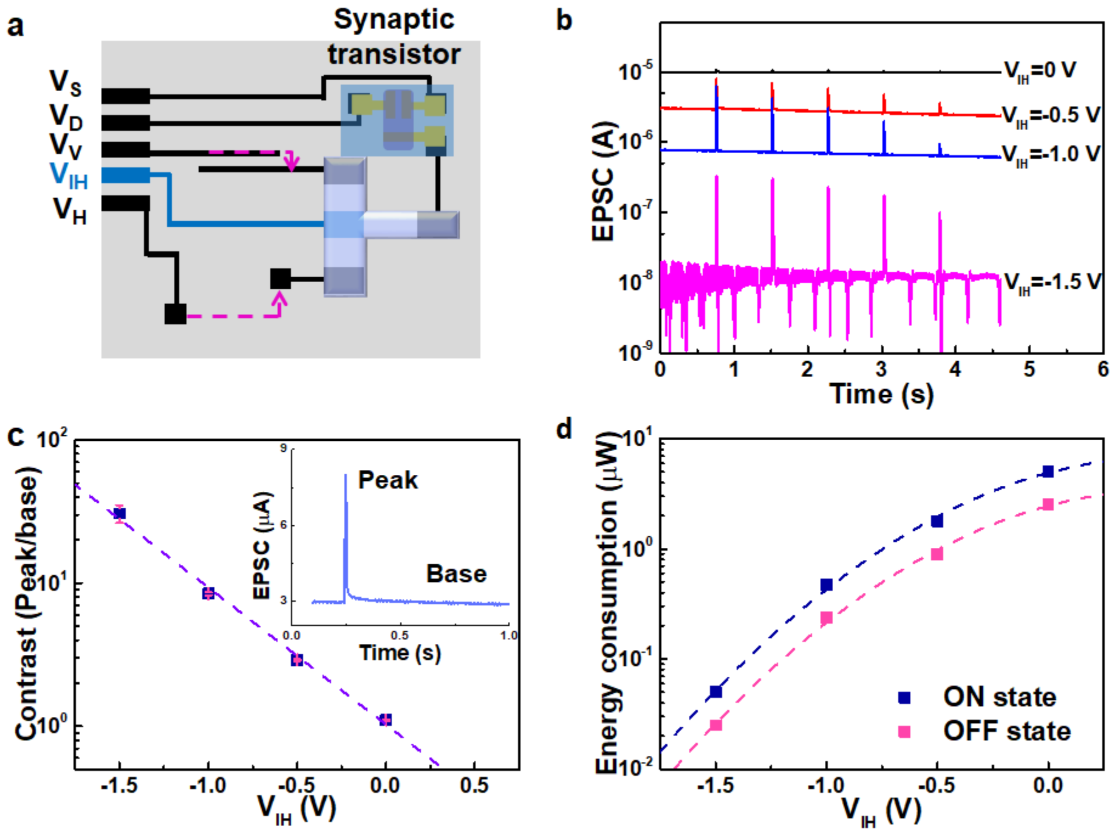

Supplementary Figure 7: The energy consumption modulation by the global inhibitory input (V_{IH}). a, The schematic diagram illustrating the global inhibitory input (V_{IH}). **b,** The EPSC responses with different inhibitory inputs (0, -0.5, -1.0, and -1.5 V, respectively). **c,** The contrast (peak/base ratio) versus different voltage applied on the inhibitory input. The inset: a typical EPSC response by a normal input (1.0 V). **d,** The power consumption of the devices with different inhibitory inputs under ON/OFF state. ON (OFF) state refers to the state with stimuli on (off).

Biological sensory system relies on powerful neural network that homeostatically or globally regulate synaptic strengths (i. e. homeoplasticity) to enable high sensitivity and to avoid destabilizing the system at ultralow energy expense.[3] Inspired by this, we introduce a global inhibitory input to the ionic/electronic hybrid neural circuit to

address the energy issue. The presynaptic voltage pulses were applied on a voltage supply of a sensor (either V_V or V_H) with this pathway short-circuit, and the inhibitory inputs were applied on V_{IH} as shown in supplementary Figure 7a. The signal contrast was defined as the ratio between the base and peak of the EPSC of the synaptic transistor. Interestingly, the contrast increases with decreased inhibitory voltages (supplementary Figure 7b and 7c). This phenomenon could be interpreted by different modification effects on the value of base and peak by the inhibitory inputs. The peak value is the sum of the base value and the amplitude of EPSC. The amplitude of EPSC is mainly due to the shift in the threshold voltage (V_{TH}) by the inhibitory input (that is applied on the gate), while the base value is dependent on the subthreshold slope (SS) of the transistors.[4] Whereas the V_{TH} can be tuned by V_{IH} within several folds, the base value can be tuned several orders of magnitude (transfer curves in supplementary Figure 7b). As a result, the peak value decays much slower than the base value with a decrease in inhibitory voltage. The power consumption with different inhibitory voltages herein is plotted in supplementary Figure 7d. Such results indicate the power consumption (<0.05 μW) be almost 2 orders of magnitudes lower than recently reported artificial sensory nerves with the lowest inhibitory voltage (-1.5 V) used in this work.[2, 5]

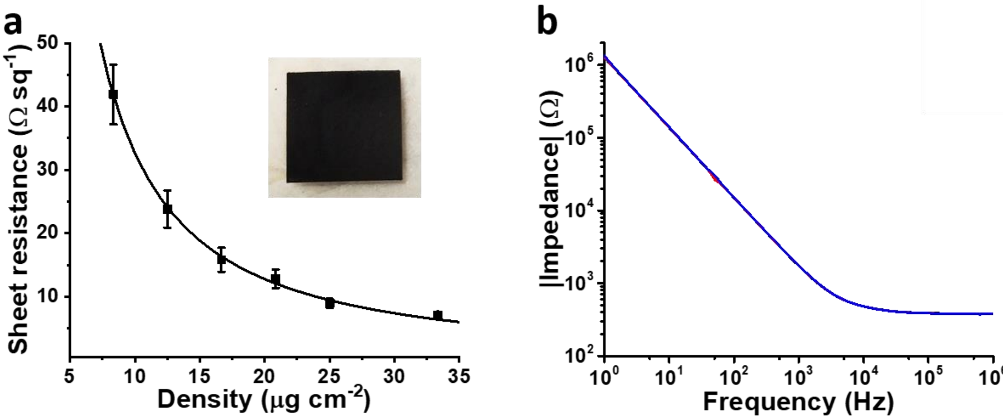

Supplementary Figure 8: Characterization of the PFT patterned CNT electrodes.

a, The sheet resistance plotted as a function of the CNT density. **b,** The impedance plotted as a function of frequency.

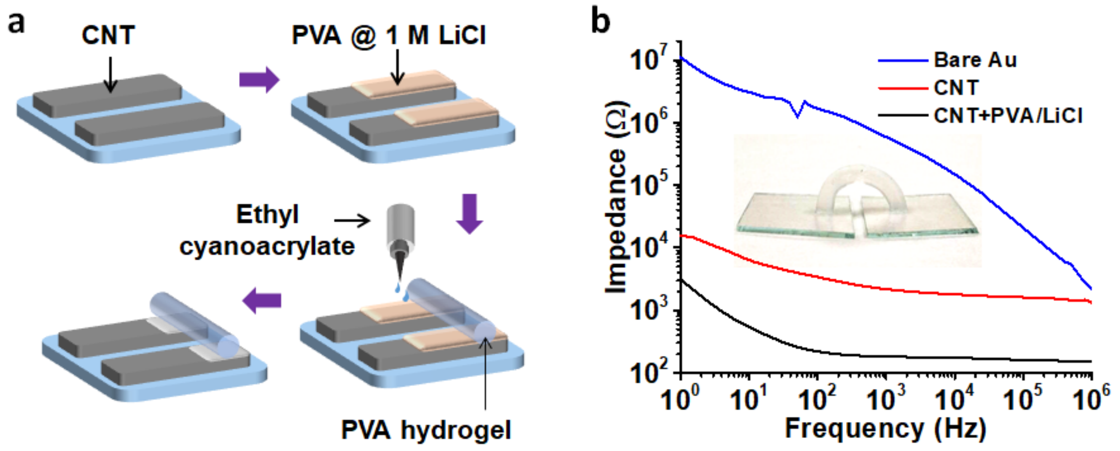

Supplementary Figure 9: The fabrication and characterizations of the adherent PVA based hydrogel on electrode. a, The fabrication steps of adhesion. **b,** The impedance-frequency curves for bare gold, CNT, and CNT+PVA/LiCl electrodes, respectively.

1. The adhesion process

The adhesion process is modified from ref. [6]. In brief, drops of 5% PVA in 1 M LiCl solution were dropped on the CNT electrodes (or other electrodes (e. g. Au and ITO) with CNT film on them). The PVA hydrogel was then placed on the PVA drops for 10 min. The ethyl cyanoacrylate (Loctite 406, Henkel) was used for bonding the PVA hydrogel and the CNT electrodes as shown in supplementary Figure 9a. The bonding occurs within seconds as the water (hydroxide ions) in the hydrogel initiates and accelerates the polymerization process of the cyanoacrylate adhesives.

2. The electrical characterizations

The impedance of the PVA after bonding on bare Au electrodes, CNT electrodes with and without PVA and salt solution droplets, respectively, are shown in supplementary Figure 9b. The CNT electrodes with PVA and LiCl droplets exhibit the lowest

impedance. The total impedances between two opposite electrodes can be simplified as two parts: the impedance of PVA hydrogel and the interfacial impedance between the hydrogel and electrodes. As the impedance of the PVA hydrogel itself is assumed to be consistent between samples, the CNT electrodes with PVA and salt solution droplets show the lowest interfacial impedance between PVA hydrogel. Such result indicates the PVA and salt solution can significantly reduce the interfacial impedance by using ethyl cyanoacrylate to bond the PVA hydrogel to conducting materials.

The impedance measurements were performed by Solartron 1260A Impedance/Gain-Phase Analyzer with a humidity of 55% RH.

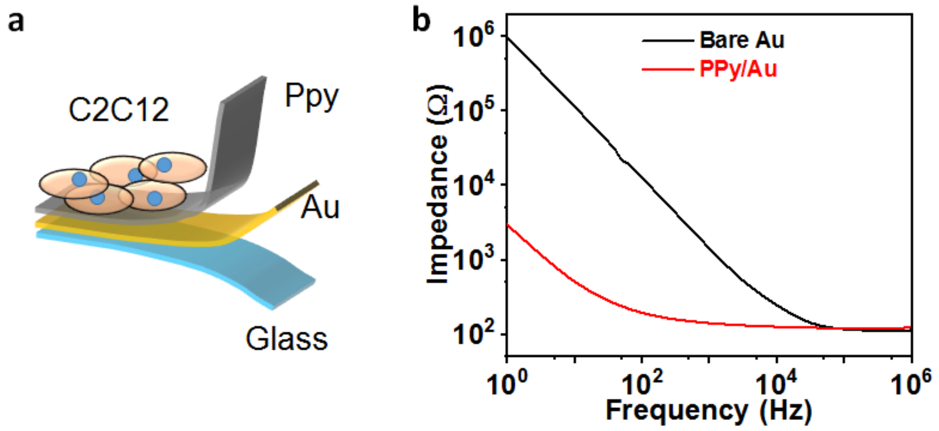

Supplementary Figure 10: The characterization of PPy/Au bioelectrodes for biohybrid neuromuscular junction. a, The schematic diagram of the PPy/Au electrode. **b,** The impedance comparison of bare gold and PPy/Au electrodes. The impedance was measured through a drop of PBS solution using Solartron 1260A Impedance/Gain-Phase Analyzer. The effective area of the electrode is 30 mm^2 with a distance of 2 mm.

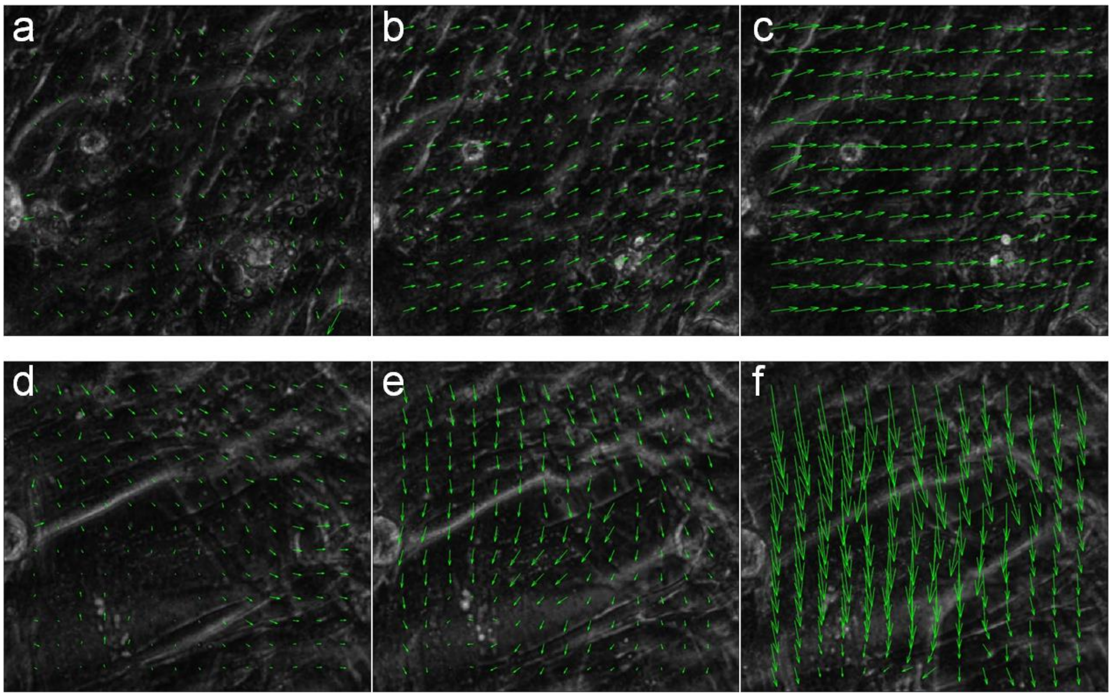

Supplementary Figure 11: Representative mapping of the velocity vector in **a-c** Region I and **d-f** Region II, under stimuli of 0.2 V, 0.6 V and 1.0 V bias, respectively. The direction of electric field in Region I and Region II are from right to left and from top to bottom.

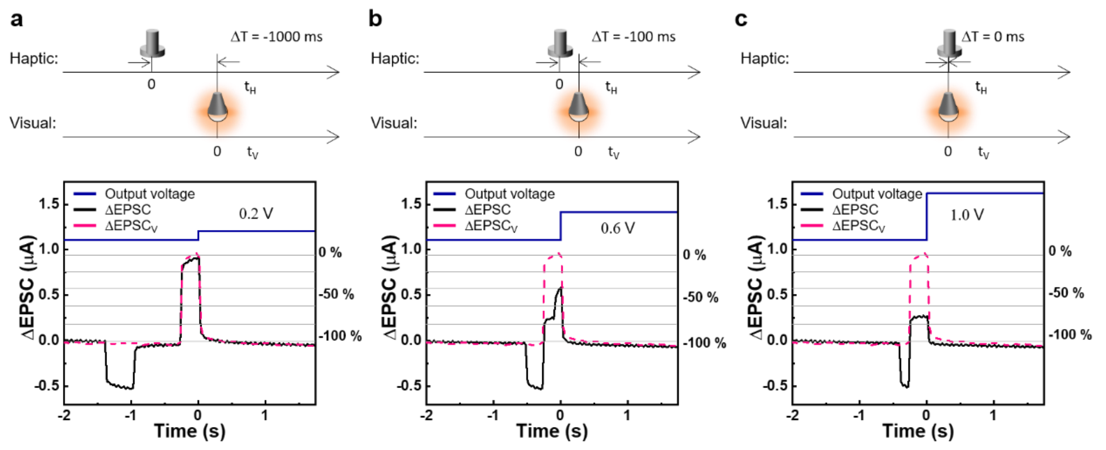

Supplementary Figure 12: **The output voltage in response to different ΔEPSC%.** When the time interval between visual and haptic stimulus are **a** -1000 ms, **b** -100 ms, and **c** 0 ms, the ΔEPSC% are estimated to be -4%, -41%, and -76%, and the output voltages are 0.2, 0.6, and 1.0 V, respectively. The intensity of light and pressure are 4.8 mW cm^{-2} and 2 kPa as mentioned in the main text.

The relative change of ΔEPSC against ΔEPSCv ((ΔEPSC-ΔEPSC$_V$)/ΔEPSC$_V$ and annotated as ΔEPSC%, where ΔEPSC$_V$ is the EPSC amplitude triggered by visual stimulation only) obtained from the BASE patch is detected and converted to voltage based on the following equation:

$$U = \frac{\Delta EPSC\%}{\max(\Delta EPSC\%) - \min(\Delta EPSC\%)} \cdot (V_{max} - V_{min}) + V_{min} \qquad 1)$$

where V_{max} and V_{min} are the maximum and minimum voltages applied on the BNJ, respectively.

In this work, the maximum and minimum ΔEPSC are ~80% and 0%, respectively, as shown in Fig. 3g, and the maximum and minimum applied voltages are 1.0 V and 0.2 V, respectively.

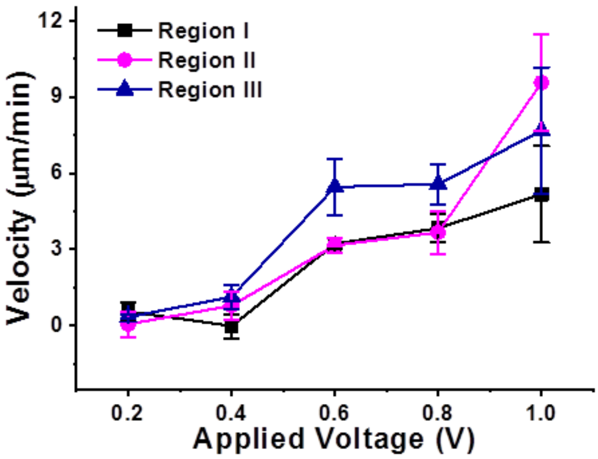

Supplementary Figure 13: The electrical characterizations on the skeletal myocytes.

The voltage applied on the biohybrid neuromuscular junction (BNJ) is provided by a programmable voltage source. The voltage is continuously applied on BNJ for 3 min for each voltage value. We estimated the velocity of the dominant direction (as demonstrated in Fig. 3d) by using the particle image velocimetry (PIV) analysis. Four frames are selected for analysis for each case, representing the state of the cell as t=0, 1, 2, and 3 min, respectively. We then applied the PIV analysis by comparing every two neighbor frames. The velocity of the dominant direction is denoted by the mean value of all velocity vectors in each frame. In this case, there are three datasets for each voltage value. The voltages used in this Figure are 0.2, 0.4, 0.6, 0.8 and 1.0 V, respectively. The three regions (as demonstrated in Fig. 3d) show similar trend that the cells start to trigger a movement when output voltage U>0.4 V.

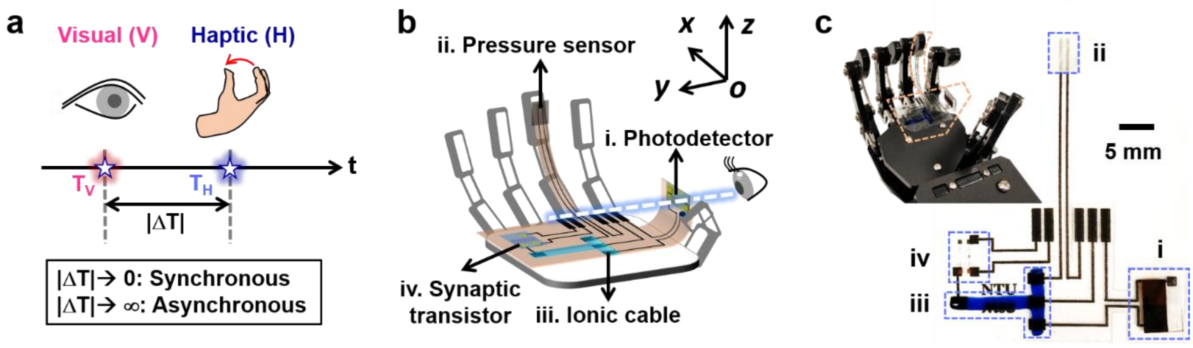

Supplementary Figure 14: The BASE based visual-haptic fusion for grasp guidance. a, The schematic diagram to illustrate the synchronization in eye–hand coordination. **b,** The schematic diagram for demonstrating the details of the BASE patch on the robotic hand. **c,** Digital images of the modified BASE patch on a robotic hand (left) and on a flat substrate (right). i to iv are photodetector, pressure sensor, ionic cable, and synaptic transistor, respectively.

Similar to the experiment of innervating muscle fiber contraction, the localization of an object at close proximity for a grasp action could be divided into two sequential steps: 1) checking by eye (t=T_V) and 2) exploring by hand (t=T_H). (**Supplementary Fig. 14a**). A modified configuration of BASE was developed by integrating visual stimuli next to the palm and haptic stimuli on the middle finger fingertip (**Supplementary Fig. 14b** and **14c**). In this case, the photodetector is analogous to the visual receptor in the eye and the pressure sensor is analogous to the mechanoreceptor under the skin of a middle finger.

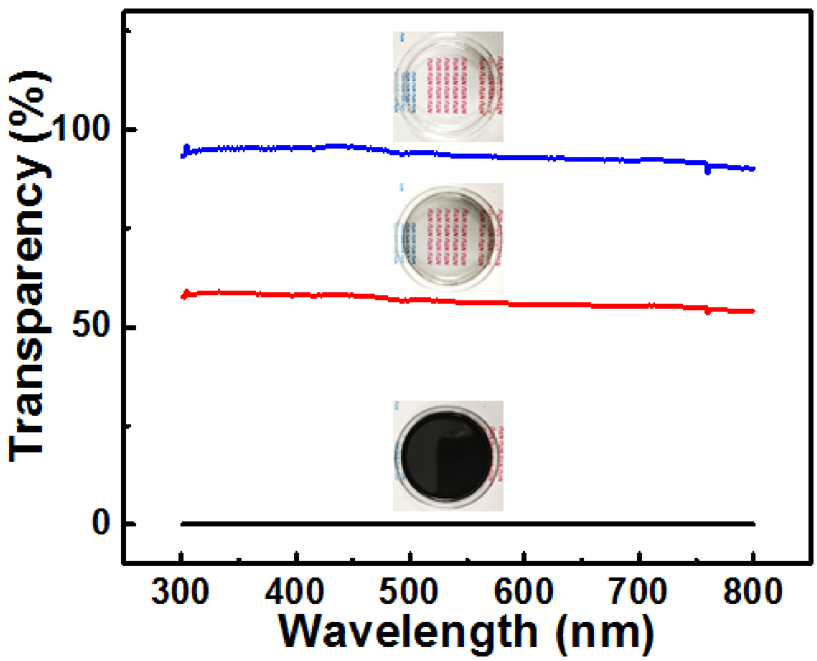

Supplementary Figure 15: The fabrication and characterizations of three PDMS patterns with different transparency. The wavelength of the test light was swept from 300 to 800 nm. The insets show the three PDMS disks with different transparency (transparent (93.4% transmittance @ 550 nm), gray (56.3% transmittance @ 550 nm), and black (0% transmittance @ 550 nm), respectively). These patterns were used to obtain the integration properties by the BASE as demonstrated in Figure 4.

The fabrication processes of the patterns are described as follow. Firstly, 2 mL hexane (Sigma-Aldrich) was stirred and mixed with carbon black powder (Sigma-Aldrich). The mixed solutions were then stirred to obtain an apparent homogenous mixture. Next, the mixtures of PDMS elastomer and crosslinker (Sylgard 184, Dow Corning) in 10:1 (w/w) ratio were added into the carbon black/hexane mixtures. The mixtures were cured at 90 °C for 2 h, then the films were peeled off from the mold. The mass

ratio between carbon black and PDMS are 0, 1:4000 w%, 1:15 w% for the transparent, gray and black patterns, respectively.

The transparency of the PDMS patterns were measured by UV-VIS spectrophotometer (SHIMADZU Co. Ltd.).

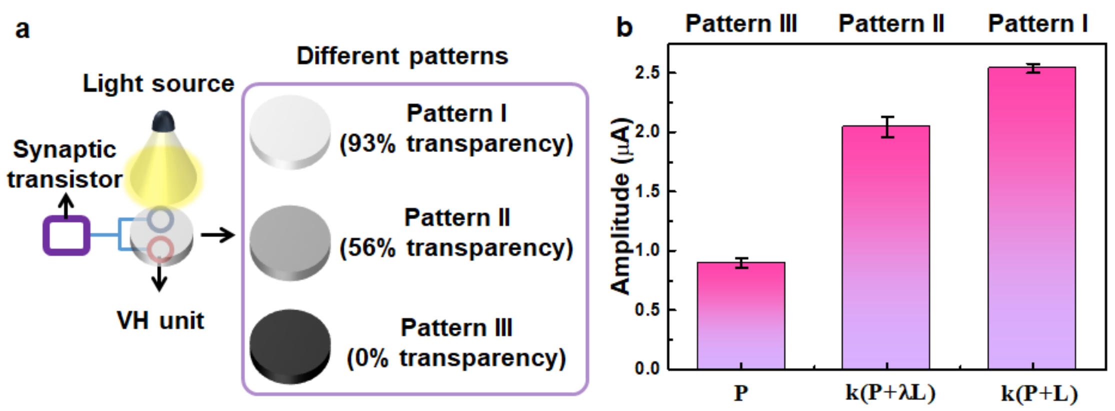

Supplementary Figure 16: Three types of multi-transparency patterns to trigger a combined response by the BASE. a, Three kinds of patterns and the BASE with one visual & haptic (VH) unit (a pressure sensor and a photodector connected to the synaptic transistor through a 'Y' shape ionic cable, and the sensors have a same distance of 2 mm (D_0) to the synaptic transistor through the cable). The patterns were put on the VH unit for ~1.5 s with light (~4.8 mW cm^{-2}) on at the same time. The transparency of the three patterns are 93 %, 56 %, and 0 %, respectively, which were fabricated by using the method described in supplementary Figure 15. The size of the pressure sensor is 1×1.25 cm^2 and the thickness of the PDMS pattern is ~2 cm (density of PDMS is ~9.65 g cm^{-3}). Therefore, the patterns would induce ~2 kPa pressure on the pressure sensor. **b,** The responses to the three kinds of the patterns, respectively. The PDMS pattern was placed on the VH unit with the light on for 1.5 s and then moved away with the light off.

1) The transparent one (Pattern I) can trigger a joint response by both pressure stimuli and light stimuli and the amplitude is estimated as:

$$AI=k\cdot(P+L) \qquad 2)$$

where P and L are the output amplitude triggered by individual pressure sensor and photodetector, respectively. The parameter k is a coefficient for multi-input integration of the synaptic transistor and is discussed in details in Supplementary Fig. 19.

2) The gray one (Pattern II) can also trigger a joint response and the amplitude is estimated as:

$$AII = k\cdot(P+\lambda L) \quad 3)$$

where λ is the proportionality coefficient ranged 0~1 depending on the light intensity passing through the pattern II.

3) The black one (Pattern III) can provide only pressure stimuli to the VH unit and the amplitude is estimated as:

$$AIII = P \quad 4)$$

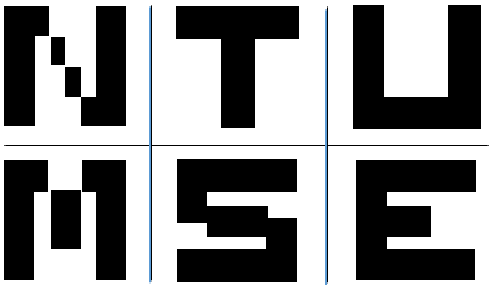

Supplementary Figure 17: The six basic alphabetic patterns: "NTUMSE". Each basic pattern was extended with three transparencies: transparent, translucent, and opaque. Each pattern was added with 5 noise pixels (40 combinations) as one multi-transparency pattern for recognition task. The distribution and the transparency of noise pixel were generated by MatLab. As such, there are 720 multi-transparency patterns in total.

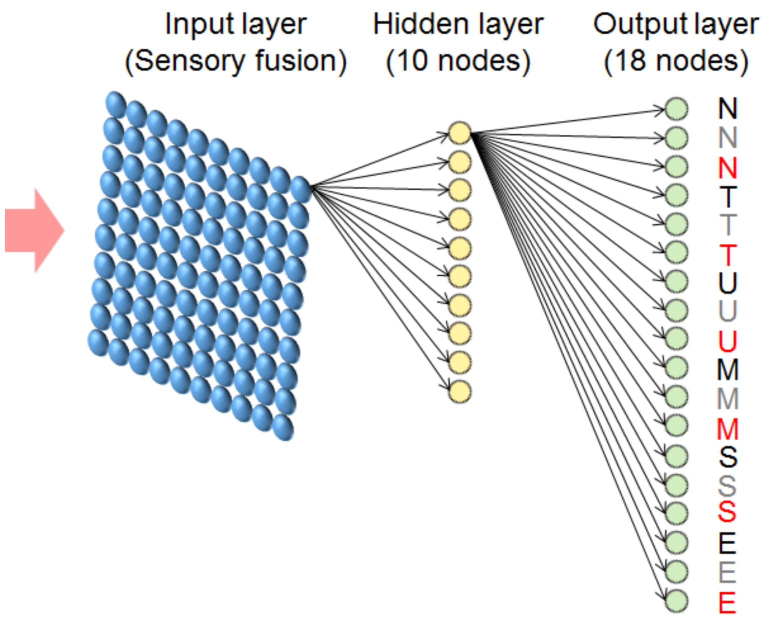

Supplementary Figure 18: Schematic diagram of the perceptron for recognition task. The data obtained from the sensory fusion matrix was fed into the two layers of perceptron. There are 10 nodes in the hidden layer and 18 nodes in the output layer corresponding to the 18 different patterns.

The recognition process was performed by MatLab toolbox. The parameters of the artificial neural network built by MatLab are listed as follow:

Number of nodes	Training algorithms
10	logsig
18	purelin

The parameters for training and display is listed as follow:

net.trainparam.show = 80; % period for results display

net.trainparam.epochs = 5000; %maximum learning epochs

net.trainparam.goal = 0.001 ; % target error

net.trainParam.lr = 0.01 ; % Learning rate

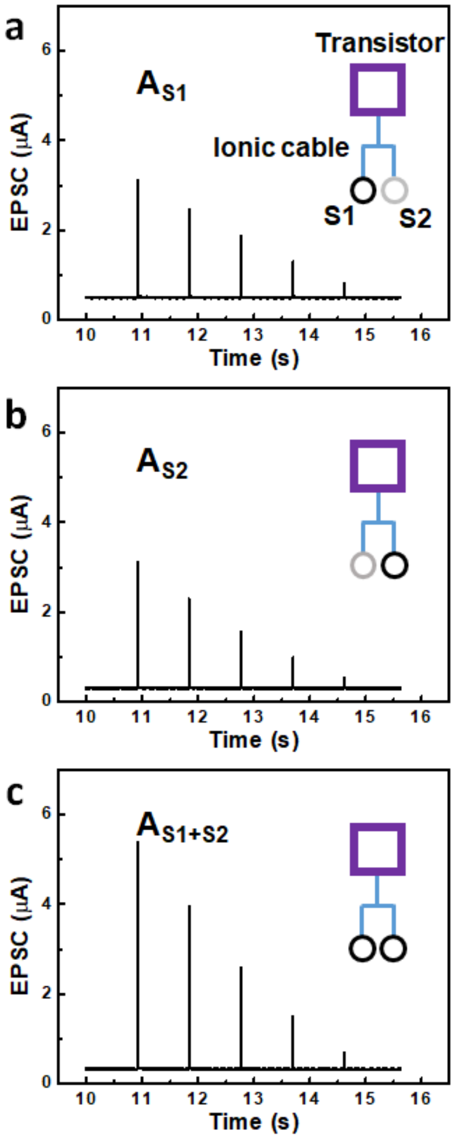

Supplementary Figure 19: **Spatial integration by the synaptic transistors.** The voltage pulses were applied on **a,** input S1 and **b**, S2 individually, and on **c**, S1 and S2 simultaneously, respectively. The amplitudes of the voltage pulses were 1.0, 0.8, 0.6, 0.4, and 0.2 V, respectively. The amplitudes of the output (A_{Si} or A_{S1+S2}, i=1 or 2) were measured by applying a fixed bias of 0.5 V between drain and source electrodes.

The expected sum is the arithmetic sum (A_{S1}+A_{S2}) of the EPSCs by individually triggering the two inputs, and the measured sum is the EPSC by simultaneously

triggering the two inputs (A_{S1+S2}). Here, we define '$\widehat{A_{S1}+A_{S2}}$' as the measured sum by two spatial isolated inputs. The A_{S1+S2} was plotted as a function of $A_{S1}+A_{S2}$ in Figure 4c and shown a sublinear relationship:

$$\widehat{A_{S1}+A_{S2}} = k \cdot (A_{S1} + A_{S2}) \qquad 5)$$

where $k \approx 0.9626$ was estimated from the curve in Figure 4c.

The parameters of P, L, and λ can then be obtained from Supplementary Figure 16b where the EPSC amplitudes triggered by the three kinds of patterns, and they were shown in Supplementary Table 3.

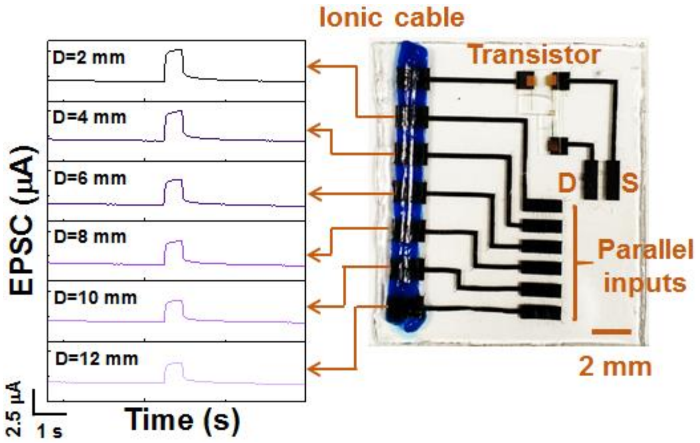

Supplementary Figure 20: The distance dependent properties of the parallel inputs connected to the synaptic transistor through an ionic cable. The left part shows the EPSCs triggered by the parallel inputs with different distance to the synaptic transistors. The right part shows the digital image of the circuit for measuring the distance dependent properties.

The EPSC amplitude (A) triggered by the input with distance of D can be fitted well by an empirical equation:

$$A=(A_z-A_\infty)\cdot D^\beta+A_\infty \qquad 6)$$

where A_z, A_∞, and D are amplitude with distance of 1 and infinity, and the distance to synaptic transistor, respectively, and β are fitting parameters. If $A_z>>A_\infty$, the aforementioned equation could be simplified to: $A\approx A_z\cdot D^\beta+A_\infty$.

In Figure 4d, the parameters A_z, β, and A_∞ can be estimated to 4.21×10^{-6}, -0.277, and 1×10^{-12} (**Supplementary Table 4**). The amplitude can be simplified to: $A=A_z\cdot D^\beta$ in this case. We also define the unit length D_0 as 2 mm in this work. The amplitude can be further transformed as:

$A = A_0 \cdot (D/D_0)^{\beta}$ 6.1)

where A_0 is the amplitude triggered by the same input with a distance of D_0 to the synaptic transistor. Here we define the $(D/D_0)^{\beta}$ as the weight of the input with distance of D:

$w = (D/D_0)^{\beta}$ 7)

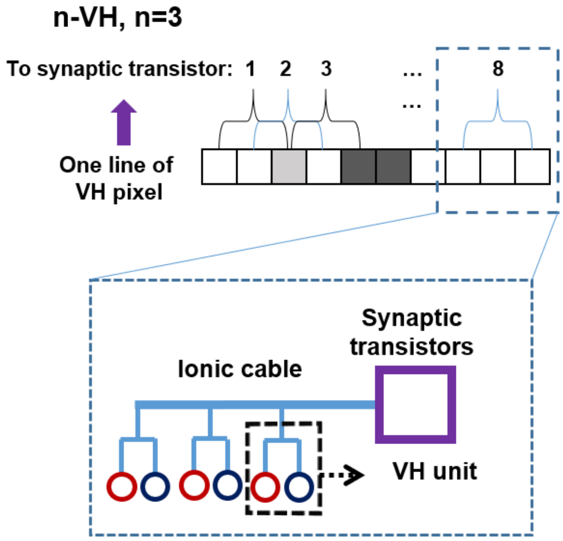

Supplementary Figure 21: Schematically illustrating the integration with the kernel size *n* (denoted by: *n*-VH fusion) by the synaptic transistors. For *n*-VH fusion, the synaptic transistor *i* (*i*=*1*, *2*, *…10-n+1*) integrates the sensory data VH units numbered from *i* to *i*+*n-1* (*e. g.* n=3 in this figure).

We assume all transistors have the same parameters. In this case, the amplitude of a transistor with kernel size n can be estimated by:

$$A_i = \sum_{m=i}^{N} f(V_m, H_m) \cdot w(N - m + 1) \qquad 8)$$

where *N*=*n*+*i*-1 and *w* is the weight of the input as demonstrated in Eq. 7.

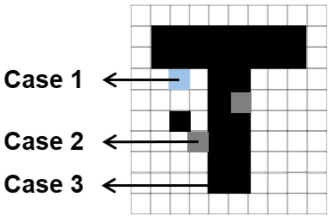

Supplementary Figure 22: Three cases for a VH unit when the detection was conducted by the sensory fusion matrix: transparent pixel (in light blue, case 1), translucent pixel (in gray, case 2) or opaque pixel (in black, case 3) of the pattern was placed on the VH unit. The distance for a VH unit to the synaptic transistor is D_i.

The output amplitude for each case can be calculated as follow:

Case 1: As the transparency of the blue pixel is ~93%, there is almost no light change compared to the background. The VH unit can only detect the pressure. Therefore, the contribution from this VH unit can be estimated from Eq. 4 and Eq. 6:

$$A=\mathrm{P}\cdot(D_i/D_0)^{\beta} \qquad 9)$$

Case 2: The VH unit could detect both light and pressure change for the gray pixel. Therefore, the contribution from this VH unit can be estimated from Eq. 3 and Eq. 6:

$$A\approx k\cdot(P+\lambda L)\cdot(D_i/D_0)^{\beta} \qquad 10)$$

Case 3: As the transparency of the black pixel is ~0%, the corresponding VH unit could only detect the pressure change when the pattern was placed on the array with the light on. Therefore, the contribution from this VH unit can be estimated from Eq. 2 and Eq. 6:

$$A\approx k\cdot(P+L)\cdot(D_i/D_0)^{\beta} \qquad 11)$$

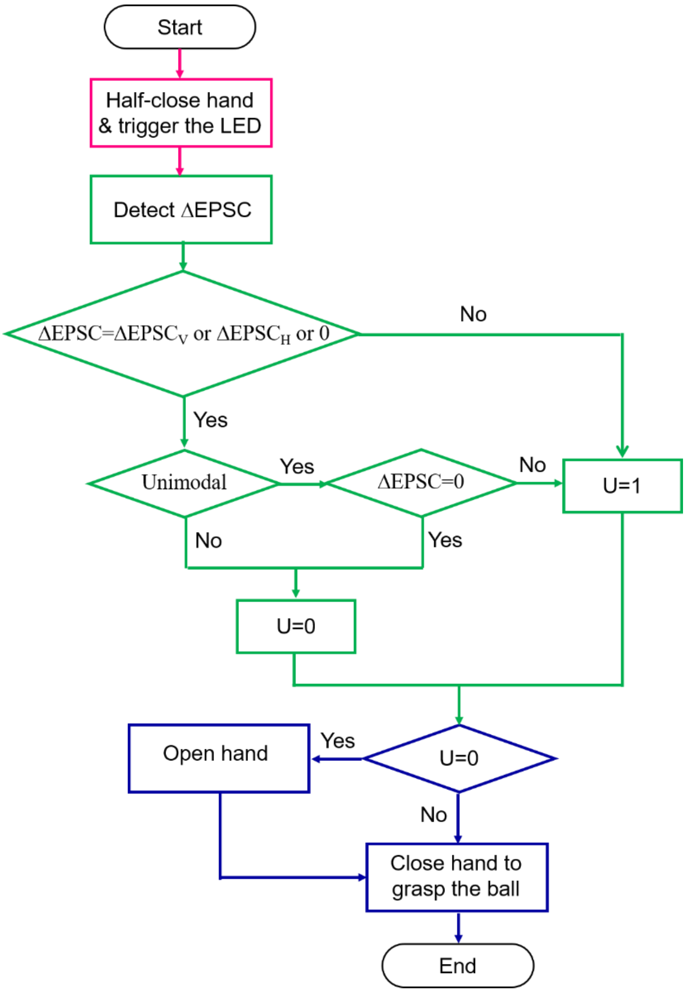

Supplementary Scheme 1: **The flowchart of the exploration (in pink)-sensing (in green)-action (in red) process for the robotic hand to grasp a tennis ball.**

To implement BASE in the grasp-guidance experiment, a robotic hand was programmed to perform the aforementioned two-step localization of the tennis ball. Step 1 was recapitulated by the photodetector that "checked" the visual stimulus from a LED attached on the ball (~4.8 mW cm^{-2}, ~250 ms). Step 2 was recapitulated by the pressure sensor that "explored" the haptic stimulus (~2 kPa, ~350 ms) from the ball by bending the middle finger to touch it. The herein obtained *ΔEPSC* was used for the decision-making, which was correspondingly converted to voltage outputs (*U*) to

trigger hand closure (U=1) or hand opening (U=0). As demonstrated in the flowchart (**Supplementary scheme 1**), the whole process can be summarized as '*explore-interpret-act*'.

	xoz	xoy
***P**osition of* ***V**isual* ***I**llusion*	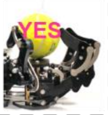	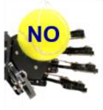
***P**osition of* ***H**aptic* ***I**llusion*	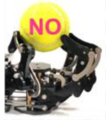	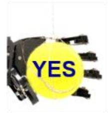
***P**osition of* ***R**ight* ***P**lace*	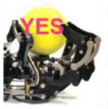	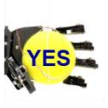

Supplementary Table 1: The three typical positions of the tennis ball located at the proximity of a robotic hand.

The positions of the tennis ball before the grasp action were set as position of right place (PRP), position of visual illusion (PVI), and position of haptic illusion (PHI). The tennis ball was noted as “YES” (or “NO”) along y-axis or z-axis, if it could (or not) deliver either the visual or haptic feedback, respectively. The ball cannot be grasped, if the ball is on the palm but could not be reached by the middle finger (PVI, V=YES and H=NO) or if the ball is not on the palm but still could be reached by the middle finger (PHI, V=NO and H=YES). Only if the ball is on the palm and its centre is aligned with the middle finger (PRP, V=YES and H=YES), it can be grasped by the robotic hand.

a	ΔEPSC	Visual neuron	Result
PVI	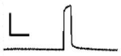 ΔEPSC$_V$	U=1 CLOSE 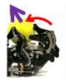	Ball slipped off (misjudgement)
PRP	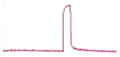 ΔEPSC$_V$	U=1 CLOSE 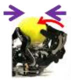	Held the ball
PHI		U=0 OPEN 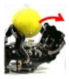	

b	ΔEPSC	Haptic neuron	Result
PVI	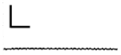	U=0 OPEN 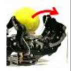	
PRP	ΔEPSC$_H$ 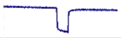	U=1 CLOSE 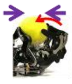	Held the ball
PHI	ΔEPSC$_H$ 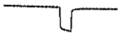	U=1 CLOSE 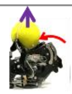	Ball slipped off (misjudgement)

c	ΔEPSC	Bimodal neuron	Result
PVI	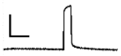 ΔEPSC$_V$	U=0 OPEN 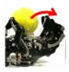	
PRP	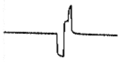	U=1 CLOSE 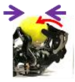	Held the ball
PHI	ΔEPSC$_H$ 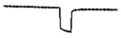	U=0 OPEN 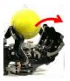	

Supplementary Table 2: The grasp-guidance experiments based on three types of artificial sensory neuron (**a**, visual type; **b**, haptic type; **c**, the bimodal type)**.** The 'V', 'T', and 'H' in the 1st row of each sub-table denote the photodetector, synaptic transistor, and pressure sensor, respectively. For each case, if U=0 (U=1) the robotic hand would open (close) based on the procedure demonstrated in **Supplementary Scheme 1**. The red and purple arrows indicate the direction of the finger movements and the ball's moving direction, respectively. The ΔEPSCs (the scale bar: 1 mA for y-axis and 1 s for x-axis) were measured from each type of the artificial sensory neuron with V_V=1.0 V, V_H=-1.0 V. Time interval between the two stimulus is 100 ms. The

ball can be grasped when the it is at PRP as U=1 for all three cases. However, when the ball is at PVI and PHI for the artificial visual and artificial haptic neuron respectively, U is also 1. The robotic hand would therefore close but the ball would slip off.

The three scenarios of decisions are summarized in **Supplementary Table 2**. It is noteworthy that all the PRP, PVI, and PHI can be differentiated by the bimodal type neuron BASE. However, either artificial visual neuron or artificial haptic neuron (corresponding to the BASE with only visual (or haptic) sensory channel activated, respectively) would generate only $\Delta EPSC_V$ (or $\Delta EPSC_H$) during the explore-interpret process when the ball was at both the PRP and at the PVI (or PHI), respectively. As a result, unimodal sensory feedback alone would lead to misjudgements and failure of catching the ball.

Supplementary Table 3: The parameter extracted from Supplementary Figure 16.

**P**	**L**	**λ**
0.8955×10^{-6}	1.7409×10^{-6}	0.7059

Supplementary Table 4: The parameters extracted from Figure 4.

A_Z	β	A_∞
4.21×10^{-6}	-0.277	1×10^{-12}

Supplementary Note 1: Calculation for the integration results.

1) The 1-VH fusion.

As all the inputs have a same distance (D_0) to the synaptic transistor, the fusion results by each pixel can be estimated by:

$A_{case1} \approx P$ 12-1)

$A_{case2} \approx k \cdot (P + \lambda L)$ 12-2)

$A_{case3} \approx k \cdot (P + L)$ 12-3)

2) The n-VH fusion.

As demonstrated in Supplementary Figure 20, the synaptic output from spatial isolated inputs that are connected to synaptic transistor through one common ionic cable is distance dependent: $A \approx A_z \cdot D^{\beta}$.

Here, we define '$\widehat{A1+A2}$' as the measured sum by two spatial isolated inputs. The multiple inputs with different distances can be deduced as:

$$\widehat{A1+A2} = k \cdot (A1 + A2) \Rightarrow A1 + \widehat{A2 \cdots} + An = k^{n-1}(A1 + A2 \cdots + An) \quad 13)$$

Thus the sensory data integrated by the transistor i could be estimated as:

$$A_{n-VH}^{i} \approx k^{n-1}(A_i\,(D_i/D_0)^{\beta} + \cdots + A_{i+n-1}(D_{i+n-1}/D_0)^{\beta} = k^{n-1} \cdot \sum_{i}^{i+n-1} A_s \frac{D_s}{D_0},$$

where $\mathrm{i} = 1,2 \ldots 10 - \mathrm{n} + 1$ 14)

The center to center spacing of two VH units are set to 3 mm, and the distance for the first VH unit to the tenth VH unit [$D_1, D_2, \ldots D_{10}$] is assumed to be [1, 4, … 31] mm.

Supplementary Method:

The characterization of the CNTs/PDMS based pressure sensors: The size of the pressure sensor is 1×1.25 cm^2. For characterization of the CNTs/PDMS based pressure sensors, pressure was applied and measured by a motorized vertical test stand (Mark-10 ESM301) in combination with a force gauge (Mark-10 M5–2). The electrical measurements were performed on a semiconductor parameter characterization system (Keithley 4200 SCS).

Sheet resistance measurements: The sheet resistance of the patterned CNT was measured through four-probe (resistivity measurement system, CMT-SR2000N) measurements. Each data point was obtained from four samples. The lowest sheet resistance of 7.0 Ω sq^{-1} was obtained with a CNT density of 33 μg cm^{-2}. The density (σ) is calculated by: $\sigma = V \cdot C / S$, where V, C, and S are the volume and the concentration of the CNT solution for vacuum filtration, and the area of the electrodes, respectively. In this work, the concentration of the CNT solution is 1 mg mL^{-1}. The inset shows the electrode sample for four-probe measurement and the size is 10 × 10 mm.

Impedance measurements: The impedance measurements of patterned CNT were characterized by a Solartron 1260A Impedance/Gain-Phase Analyzer. The impedance was measured through covering a drop of phosphate buffered saline (PBS) on a patterned CNT electrode-pairs with area of 0.15 cm^2 and gap-width of 1.25 mm.

Supplementary References

1. Zhu B., et al. Microstructured Graphene Arrays for Highly Sensitive Flexible Tactile Sensors. *Small* **10**, 3625-3631 (2014).

2. Wan C., et al. An Artificial Sensory Neuron with Tactile Perceptual Learning. *Adv Mater* **30**, 1801291 (2018).

3. Gkoupidenis P., Koutsouras D. A. & Malliaras G. G. Neuromorphic device architectures with global connectivity through electrolyte gating. *Nat Commun* **8**, 15448 (2017).

4. Wan C., Zhu L., Wan X., Shi Y. & Wan Q. Organic/inorganic hybrid synaptic transistors gated by proton conducting methylcellulose films. *Appl Phys Lett* **108**, 043508 (2016).

5. Kim Y., et al. A bioinspired flexible organic artificial afferent nerve. *Science* **360**, 998-1003 (2018).

6. Wirthl D., et al. Instant tough bonding of hydrogels for soft machines and electronics. *Sci Adv* **3**, e1700053 (2017).
