# Supplementary material for: An artificial sensory neuron with visual-haptic fusion
Source: Nat Commun. 2020 Sep 14;11:4602. doi: 10.1038/s41467-020-18375-y (PMC7490423; doi:10.1038/s41467-020-18375-y)
Supplement: Supplementary file 2 — Description of Additional Supplementary Files [file 41467_2020_18375_MOESM2_ESM.docx]

Description of Additional Supplementary Files

Title: Supplementary Movie 1-3

Description: The myotubes contraction triggered by BASE with time interval of -1000, -100, and 0 ms between the visual and haptic stimulations, respectively.

Title: Supplementary Movie 4 and 5

Description: The ball is at place of visual illusion (PVI) with artificial visual neuron and bimodal neuron activated, respectively.

Title: Supplementary Movie 6

Description: The ball is at the place of right place (PRP) with artificial bimodal neuron activated, respectively.

Title: Supplementary Movie 7 and 8

Description: The ball is at place of haptic illusion (PHI) with artificial visual neuron and bimodal neuron activated, respectively. The time intervals of the two-step exploration (i.e. the difference of triggering time between photodetector and pressure sensor) are around ±500 ms in all cases. The difference is due to the mismatch between the robotic hand actuation time and the triggering of the LED.
